# Postoperative Complications and Swallowing Function after Jejunal and Skin Flap Reconstruction for Hypopharyngeal Carcinoma—A Multicenter Retrospective Study

**DOI:** 10.3390/jcm11051464

**Published:** 2022-03-07

**Authors:** Kunihiko Tokashiki, Isaku Okamoto, Takuro Okada, Hiroki Sato, Taku Yamashita, Takashi Matsuki, Takahito Kondo, Chihiro Fushimi, Tatsuo Masubuchi, Kouki Miura, Go Omura, Kiyoaki Tsukahara

**Affiliations:** 1 Department of Otorhinolaryngology, Head and Neck Surgery, Tokyo Medical University, Tokyo 160-0023, Japan; m03075kt@tokyo-med.ac.jp (K.T.); taku-hns@tokyo-med.ac.jp (T.O.); satohiro@tokyo-med.ac.jp (H.S.); tsuka@tokyo-med.ac.jp (K.T.); 2 Department of Otorhinolaryngology-Head and Neck Surgery, Kitasato University School of Medicine, Sagamihara 252-0374, Japan; tyanahns@kitasato-u.ac.jp (T.Y.); matsuki@med.kitasato-u.ac.jp (T.M.); 3 Department of Otolaryngology, Head and Neck Surgery, Tokyo Medical University Hachioji Medical Center, Tokyo 193-0998, Japan; takajin@tokyo-med.ac.jp; 4 Department of Head and Neck Oncology and Surgery, International University of Health and Welfare Mita Hospital, Tokyo 108-8329, Japan; chihiro.fushimi@iuhw.ac.jp (C.F.); masubuchi@iuhw.ac.jp (T.M.); kmiura@iuhw.ac.jp (K.M.); 5 Department of Head and Neck Surgery, National Cancer Center Hospital, Tokyo 104-0045, Japan; goomura@msn.com

**Keywords:** free jejunal flap, cutaneous free tissue flaps, hypopharyngeal carcinoma, total pharyngolaryngectomy

## Abstract

This study compared the incidence of perioperative complications and swallowing function between free jejunal flap reconstruction and cutaneous free tissue flap construction. We included 223 patients who underwent hypopharyngeal reconstruction using free flap. At discharge, +the free jejunal flap was associated with a Functional Oral Intake Scale (FOIS) score of 1–6 in 132 cases (70%) and a score of 7 in 56 cases (30%). Regarding the cutaneous free tissue flaps, FOIS scores of 1–6 were observed in 18 cases (51%), and a score of 7 was noted in 17 cases (49%). Donor site complications occurred in 12% of the patients who underwent free jejunal flap procedures and in none of the patients who underwent cutaneous free tissue flap procedures. We found that the free jejunal flap had a regular dietary intake rate in 56 patients (30%), whereas cutaneous free tissue flaps had a regular dietary intake rate in 17 patients (49%). Cutaneous free tissue flaps had a significantly higher regular dietary intake rate at discharge and a significantly lower incidence of donor site complications than free jejunal flaps. In conclusion, free-flap reconstruction may be a better method than free jejunal flap reconstruction for the treatment of hypopharyngeal cancer.

## 1. Introduction

Head and neck cancer remains a significant public health burden, causing significant mortality and morbidity [1]. Moreover, the worldwide incidence of hypopharyngeal cancer has increased, with tobacco and alcohol consumption being its major risk factors [2]. In Japan, free jejunal flap reconstruction is recommended for the treatment of hypopharyngeal cancer [3]. However, a free jejunal flap requires laparotomy, and intestinal obstruction is a problematic complication of laparotomy [4]. Recently, cutaneous free tissue flaps have been used as reconstruction material [5]. Findings regarding the benefits of cutaneous free tissue flaps compared to free jejunal flaps are scattered across the literature [6,7]. Furthermore, some studies have demonstrated the postoperative function of cutaneous free tissue flap [8]. Our previous study on free jejunal and cutaneous free tissue flaps reconstruction in 75 patients with hypopharyngeal cancer showed no significant difference in the incidence of cervical complications between free jejunal and cutaneous free tissue flaps reconstruction (*p* = 0.99); however, we demonstrated significantly higher donor site complications in the free jejunal flap group than in the cutaneous free tissue flap group (*p* = 0.03) [9]. Notably, however, few Japanese studies have demonstrated the benefits of cutaneous free tissue flaps. Therefore, this retrospective multicenter study examined swallowing function and perioperative complications in patients who underwent hypopharyngolaryngectomy and free jejunal flap reconstruction or cutaneous free tissue flap reconstruction after the diagnosis of hypopharyngeal cancer. The purpose of this study was to clarify the benefits of cutaneous free tissue flaps with respect to postoperative complications and swallowing function after reconstruction for hypopharyngeal cancer.

## 2. Materials and Methods

### 2.1. Patients

Two hundred and thirty-five patients with hypopharyngeal cancer underwent reconstruction between 1 January 2010 and 30 April 2020 at Tokyo Medical University, Tokyo Medical University Hachioji Medical Center, International University of Health and Welfare Mita Hospital, or Kitasato University School of Medicine. After excluding 12 patients who underwent hypopharyngeal reconstruction pedicle flap, 223 patients who underwent hypopharyngeal reconstruction using free flap were included in this study. Patients who refused to participate were excluded.

### 2.2. Methods

Sex, age, medical history, subsites, T classification and N classification (The eighth edition American Joint Committee on Cancer (AJCC) staging manual) were examined. The preoperative therapy was divided into Neo-Adjuvant chemotherapy, chemoradiotherapy, and radiotherapy. Surgery was divided into definitive and salvage surgery to examine the purpose of surgery. Neoadjuvant therapy followed by surgery is included in definitive surgery. Radiotherapy and chemoradiotherapy followed by surgery are included in salvage surgery. The outcomes for swallowing function were diet at discharge, the presence or absence of nasopharyngeal reflux, and the presence or absence of anastomotic strictures. A logistic regression model was used to calculate the adjusted odds ratio (OR) with a 95% confidence interval (CI) for diet at discharge associated with the background characteristics of patients. The multivariate logistic regression model was used with adjustments for all potential confounding factors, as listed in Table 1.

Diet at discharge was evaluated using the Functional Oral Intake Scale (FOIS) classification [10]. FOIS 1-6 is tube dependent with consistent oral intake of food or liquid, and FOIS 7 is total oral diet with no restrictions. Perioperative complications were classified into the incidence of pharyngocutaneous fistula, donor site complications, systemic complications, and cervical complications. Donor site complications were divided into intestinal obstruction, neuropathy, hemorrhage, and infection. Systemic complications were classified as respiratory disorders, cardiovascular disorders, and others. Cervical complications were classified as fistulas, flap necrosis, lymph fistulas, hemorrhage, and infection. We used the surgical procedure described in detail by Tokaashiki et al. [9]. Skin incision and insetting the flap were performed by head and neck surgeons, whereas free jejunal flap reconstruction was performed by a gastroenterologist. Finally, cutaneous free tissue flap reconstruction was performed by plastic surgeons.

### 2.3. Outcomes

The primary endpoint was the swallowing function, and the secondary endpoints were the incidence of pharyngocutaneous fistula and perioperative complications.

### 2.4. Statistical Analyses

Statistical analyses were performed using Fisher’s exact test. Statistical significance was set at *p* < 0.05. All statistical analyses were performed using EZR (Saitama Medical Center, Jichi Medical University, Saitama, Japan), which is a graphical user interface for the R software environment for statistical computing and graphics (The R Foundation for Statistical Computing, Vienna, Austria). EZR is a modified version of R Commander, designed to add statistical functions frequently used in biostatistics [11]. No statistical sample size calculations were conducted. However, a sample size of 223 patients provided an ad hoc power of 51%.

### 2.5. Ethics

This study was approved by the Ethics Committee of Department of Otorhinolaryngology, Head and Neck Surgery, Tokyo Medical University, Department of Otorhinolaryngology-Head and Neck Surgery, Kitasato University School of Medicine, Department of Head and Neck Oncology and Surgery, International University of Health and Welfare Mita Hospital (approval no. T2020-0094, 5-21-36, C20-272). All protocols were conducted in accordance with the Declaration of Helsinki and written consent for treatment was obtained from each patient before surgery.

## 3. Results

### 3.1. Background Characteristics of Patients

The results of comparisons of the patients’ background characteristics between the free jejunal flap and cutaneous free tissue flap groups are shown in Table 1. Reconstruction in the 223 patients with hypopharyngeal cancer consisted of free jejunal flap reconstruction (*n* = 188) and cutaneous free tissue flap reconstruction (*n* = 35). Furthermore, cutaneous free tissue flap reconstruction comprised both anterolateral thigh flap reconstruction (*n* = 24) and forearm flap reconstruction (*n* = 11). Regarding the sex proportion, the free jejunal flap group consisted of 162 males and 26 females, whereas the cutaneous free tissue flap group comprised 31 males and four females. The mean ages of the free jejunal and cutaneous free tissue flap groups were 68 years (range, 45–84 years) and 71 years (range, 52–80 years), respectively. The pyriform sinus consisted of 133 free jejunal flaps and 25 cutaneous free tissue flaps, whereas the postcricoid region consisted of 26 free jejunal flaps and eight cutaneous free tissue flaps. Finally, the posterior wall comprised 29 free jejunal flaps and two cutaneous free tissue flaps. Regarding T classification, there were 43 T1/2 patients in the free jejunal flap group and seven T1/2 patients in the cutaneous free tissue flap group. Moreover, there were 145 T3/4 patients in the free jejunal flap group and 28 T3/4 patients in the cutaneous free tissue flap group. Regarding N classification, there were 51 N0 patients, 27 N1 patients, 92 N2 patients, and 18 N3 patients in the free jejunal flap group and eight N0 patients, six N1 patients, 18 N2 patients, and three N3 patients in the cutaneous free tissue flap group. Regarding preoperative treatment history, 26 patients in the free jejunal flap group and four in the cutaneous free tissue flap group had a history of chemotherapy. Nine patients in the free jejunal flap group and two in the cutaneous free tissue flap group had a history of chemoradiotherapy. Twenty-five patients in the free jejunal flap group and three in the cutaneous free tissue flap group had a history of radiotherapy. Regarding the purpose of surgery, patients who underwent definitive surgery consisted of 156 patients with free jejunal flap reconstruction and 31 patients with cutaneous free tissue flap reconstruction, respectively. The patients who underwent salvage surgery consisted of 32 patients who underwent free jejunal flap reconstruction and four who underwent cutaneous free tissue flap reconstruction. Age, sex, medical history, subsite, T classification, preoperative treatment history, and purpose of surgery were compared between the free jejunal flap and cutaneous free tissue flap groups. There were no significant differences in any variables between the two groups, including the patients’ background characteristics.

### 3.2. Complications

The assessment of the primary endpoint, swallowing function, is shown in Table 2.

Regarding the diet form at discharge, 132 patients had FOIS scores of 1–6 (70%), and 56 patients had a FOIS score of 7 (30%) in the free jejunal flap group, whereas 18 patients had FOIS scores of 1–6 (51%), and 17 patients had a FOIS score of 7 (49%) in the cutaneous free tissue flap group. The normal diet rate was significantly higher in the cutaneous free tissue flap group than in the free jejunal flap group (*p* = 0.04). Table 3 reports the ORs and 95% CIs from logistic regressions of diet at discharge, adjusted for the patient and setting characteristics described above.

The cutaneous free tissue flap group had a higher regular dietary intake rate than the free jejunal flap group with an adjusted OR of 0.35 (95% CI, 0.16–0.78; *p* = 0.01). Other factors, such as age, sex, medical history, T classification, preoperative therapy (neo-adjuvant chemotherapy and radiotherapy), and objective (definitive or salvage surgery) were not associated with diet at discharge.

Eleven patients in the free jejunal flap group (6%) and two patients in the cutaneous free tissue flap group (6%) had nasopharyngeal reflux. Twenty-one patients in the free jejunal flap group (11%) and three patients in the cutaneous free tissue flap group (9%) had anastomotic strictures. However, there were no significant differences between the two groups.

The assessment of the primary endpoint, swallowing function excluding radiotherapy, is shown in Table 4.

Regarding the diet form at discharge, 106 patients had FOIS scores of 1–6 (69%) and 48 patients had a FOIS score of 7 (31%) in the free jejunal flap group, whereas 13 patients had FOIS scores of 1–6 (43%) and 17 patients had a FOIS score of 7 (57%) in the cutaneous free tissue flap group. The normal diet rate was significantly higher in the cutaneous free tissue flap group than in the free jejunal flap group (*p* = 0.01). Table 5 reports the ORs and 95% CIs from logistic regressions of diet at discharge excluding radiotherapy, adjusted for the patient and setting characteristics described above.

The cutaneous free tissue flap group had a higher regular dietary intake rate than the free jejunal flap group with an adjusted OR of 0.31 (95% CI, 0.14–0.72; *p* = 0.01) Other factors, such as age, sex, medical history, T classification, and preoperative therapy were not associated with diet at discharge.

Ten patients in the free jejunal flap group (6%) and one patient in the cutaneous free tissue flap group (3%) had nasopharyngeal reflux. Twenty-nine patients in the free jejunal flap group (12%) and two patients in the cutaneous free tissue flap group (7%) had anastomotic strictures. However, there were no significant differences between the two groups.

The incidence of pharyngocutaneous fistula as the secondary endpoint is shown in Table 6.

Pharyngocutaneous fistula was observed in 21 (9%) of 223 patients. Pharyngocutaneous involvement was observed in 18 patients (10%) requiring free jejunal flap reconstruction and in three patients requiring reoperation (2%). Furthermore, it was observed in three patients requiring cutaneous free tissue flap reconstruction (9%) and in one patient requiring reoperation (3%). However, there was no significant difference between the two groups (*p* = 0.99). The data regarding perioperative complications are shown in Table 7.

Regarding donor site complications in the free jejunal flap group, 16 patients (8%) had intestinal obstruction, and six (3%) had infection. No hemorrhage or neuropathy was observed. None of the patients in the cutaneous free tissue flap group experienced donor site complications. There was no significant difference between the two groups; however, the total incidence of donor site complications was significantly higher in the free jejunal flap group than in the cutaneous free tissue flap group (*p* = 0.03). Regarding systemic complications in the free jejunal flap group, respiratory disorders were observed in five patients (3%) whereas cardiovascular disorders were observed in two patients (1%). In the cutaneous free tissue flap group, respiratory disorders were observed in two patients (6%) and cardiovascular disorders were observed in one patient (3%). However, there were no significant differences in any of the variables between the two groups. In total, 68 patients (40%) in the free jejunal flap group had cervical complications, including fistula in 24 patients (13%), flap necrosis in nine patients (5%), lymph fistula in 26 patients (14%), hemorrhage in five patients (3%), and infection in 26 patients (14%). Ten patients (31%) in the cutaneous free tissue flap group had cervical complications, including fistula in six patients (17%), flap necrosis in one patient (3%), lymph fistula in three patients (9%), and infection in three patients (9%). However, there were no significant differences in any of the variables between the two groups.

## 4. Discussion

In this study, we found that the normal diet rate was significantly higher in the cutaneous free tissue flap group than in the free jejunal flap group, whereas donor site complications were significantly higher in the free jejunal flap group than in the cutaneous free tissue flap group. Our findings indicate the benefits of cutaneous free tissue flap reconstruction on postoperative swallowing function and complications after reconstruction for hypopharyngeal cancer.

Swallowing function is the most important factor affecting the quality of life of patients after hypopharyngeal cancer reconstruction. The final goal after reconstruction is to maximize swallowing recovery and minimize the incidence of perioperative complications. Various studies have reported postoperative complications after reconstruction for hypopharyngeal cancer. Suzuki et al. [4] reported that 108 (3.3%) of 3320 patients who underwent free jejunal flap reconstruction in Japan had postoperative intestinal obstruction. They stated that older age was significantly associated with the risk of intestinal obstruction. They also insisted that the delay in oral feeding that starts 1 week after surgery, regardless of the low incidence of abdominal surgery, may increase the risk of intestinal obstruction. Razdan et al. [12] reported that two (2%) of 90 patients who underwent free jejunal flap reconstruction had postoperative intestinal obstruction. They identified free jejunal flap reconstruction as a safe method of sampling the flap, with the proficiency of the surgeon being the most important factor. When selecting a flap, it is important to consider the patient’s age, years of experience, and proficiency of the surgeon in order to minimize the risk of donor site complications. Although there may be an increased risk of cardiopulmonary insufficiency after laparotomy [7], there was no significant difference in the presence or absence of laparotomy between the two groups in this study. The incidence of cervical complications is presented in Table 8.

Peirong et al. [8] reported that the incidence of fistulas after anterolateral thigh flap reconstruction was 9%. Lewin et al. [13] reported the incidence of fistula after free jejunal flap reconstruction and anterolateral thigh flap reconstruction to be 3% and 7%, respectively. Huang et al. [14] reported the incidence of fistulas requiring anterolateral thigh flap reconstruction to be 11%. Kurita et al. [15] reported that the incidence of fistulas after free jejunal flap reconstruction was 3.7%. Furthermore, the current findings were consistent with their results. Sugiyama et al. [16] reported that the use of an open drain, cardiovascular disease, and longer operation time are significant risk factors for abscess formation, fistula formation, and cervical flap necrosis. Reconstruction with a smaller flap size has a higher possibility of fistula formation [17]. To reduce the incidence of fistulas, consideration should be given to flap size and shortening of surgery time rather than the selection of reconstruction materials. The low incidence of fistula in anterolateral thigh flap reconstruction is due to the presence of fascia, which allows double-layer suturing. Moreover, it has been suggested that the inclusion of a large muscle body also contributes to strong closure and spontaneous fistula closure [18]. It has been suggested that infection and leakage after fistula formation may lead to stenosis [19]. Therefore, evaluation of the fistula by esophagogram or esophagoscopy before oral feeding is necessary [20].

Regarding postoperative swallowing function, Peirong et al. [8] reported that 85% of patients who underwent anterolateral thigh flap reconstruction were able to consume a soft or normal diet. Lewin et al. [13] reported that the oral intake rates in free jejunal flap and anterolateral thigh flap reconstructions were 73% and 91%, respectively. In this study, the normal diet rate was significantly higher in the cutaneous free tissue flap group than in the free jejunal flap group (*p* = 0.04). Furthermore, it has been indicated that the reduced oral intake rate after free jejunal flap reconstruction due to uncoordinated swallowing and peristalsis in the esophagus may lead to nasopharyngeal reflux and dysphagia [13]. The stenosis rate after free jejunal flap reconstruction is reported to be 6% [8], and the stenosis rate after anterolateral thigh flap reconstruction is reported to be 13% [21]. In this study, there was no significant difference in nasopharyngeal reflux or anastomotic stricture between the two groups.

Our study had the following limitations: (1) small sample size, (2) all limitations and risk of bias inherent to the retrospective design, and (3) inability to generalize the findings to different populations. Future prospective studies should include the abovementioned variables to examine the difference in the oral intake rate and normal diet rate between the two groups. In addition, since this study is a retrospective study, the number of cases is biased. In Japan, free jejunal flap reconstruction is recommended for the treatment of hypo-pharyngeal cancer [3], resulting in a large number of free jejunal flap groups. Therefore, it is necessary to increase the number of cases of cutaneous free tissue flap and reexamine it in the future. Furthermore, regarding the resection range, it has been suggested that swallowing function changes depending on hypopharyngectomy (partial or total) [22,23]. This may be due to the presence of the retropharyngeal mucosa (approximately 1 cm), which prevented scar contracture. Additionally, one study indicated the necessity of measurements even if the diameter of the esophagus was ≤3 cm [23]. This study focused on the type of reconstruction material used. Future studies should also consider the resection range. Regarding speech outcomes, few hospitals in Japan provide interventions for tracheoesophageal puncture after pharyngeal reconstruction, and general awareness regarding such treatment is low. Peirong et al. [8] showed that an anterolateral thigh flap improves the effective acquisition of speech. Furthermore, a low success rate of esophageal speech after free jejunal flap reconstruction has been reported [24,25]. It would be preferable to investigate the benefits of cutaneous free tissue flap reconstruction on swallowing function and acquisition of speech as a reconstruction method for hypopharyngeal cancer.

## 5. Conclusions

We examined the benefits of cutaneous free tissue flap reconstruction as a reconstruction method for hypopharyngeal cancer on postoperative complications and swallowing function. This study demonstrated that a cutaneous free tissue flap is safer than a free jejunal flap and can be expected to improve the swallowing function at an earlier stage. Our findings suggest that cutaneous free tissue flap reconstruction is a better method for treating hypopharyngeal cancer than free jejunal flap reconstruction.

## Figures and Tables

**Table 1:** Background characteristics of patients.

	Free Jejunal Flap Group		Cutaneous Free Tissue Flap Group		*p*-Value
	N = 188		N = 35		
	N	%	N	%	
Age,: mean (range)	68.0 (45–84)		71.0 (52–80)		
Sex					
Male	162	86	31	89	0.99
Female	26	14	4	11	
Medical history					
Diabetes	30	16	7	20	0.62
Coronary artery disease	13	7	6	17	0.09
Cerebrovascular disease	16	9	2	6	0.75
Tumor subsite					
PS	133	71	25	71	0.16
PC	26	14	8	23	
PW	29	15	2	6	
T classification					
½	43	23	7	20	0.83
¾	145	77	28	80	
N classification					
0	51	27	8	23	0.94
1	27	14	6	17	
2	92	49	18	51	
3	18	10	3	9	
Preoperative therapy					
Neo-Adjuvant Chemotherapy	26	12	4	11	0.74
Chemoradiotherapy	9	5	2	6	
Radiotherapy	25	13	3	9	
Objective					
definitive surgery	156	83	31	89	0.62
Salvage surgery	32	17	4	11	

PS: Pyriform sinus; PC: postcricoid; PW: posterior wall.

**Table 2:** Postoperative swallowing function in each group.

		Free Jejunal Flap Group		Cutaneous Free Tissue Flap Group		*p*-Value
		N = 188		N = 35		
		N	%	N	%	
Diet at discharge	FOIS 1-6	132	70	18	51	0.04
	FOIS 7	56	30	17	49	
Nasopharyngeal reflux	No	177	94	33	94	0.99
	Yes	11	6	2	6	
Anastomotic stricture	No	167	89	32	91	0.99
	Yes	21	11	3	9	

FOIS: functional oral intake scale.

**Table 3:** Odds ratios (ORs) and 95% confidence intervals (CIs) from logistic regressions of diet at discharge.

	Diet at Discharge		
		Multivariable Analysis	
	N	OR (95% CI)	*p*-Value
Age			
<70	96	0.55 (0.30–1.00)	0.05
≧70	127		
Sex			
Male	193	0.64 (0.27–1.52)	0.31
Female	30		
Medical history			
Diabetes	37	1.31 (0.58–2.96)	0.51
No Diabetes	186		
Coronary artery disease	19	1.70 (0.56–5.19)	0.35
No coronary artery disease	204		
Cerebrovascular disease	18	1.72 (0.52–5.63)	0.37
No cerebrovascular disease	205		
T (TNM classification)			
½	50	1.91 (0.91–4.04)	0.09
¾	173		
Preoperative therapy			
Neo-adjuvant Chemotherapy	30	0.90 (0.37–2.20)	0.81
No neo-adjuvant Chemotherapy	193		
Radiotherapy	39	0.65 (0.28–1.51)	0.32
No Radiotherapy	184		
Objective			
definitive surgery	187	2.35 (0.91–6.06)	0.08
Salvage surgery	36		
Reconstruction material			
Free jejunal flap	188	0.35 (0.16–0.78)	0.01
Cutaneous free tissue flap	35		

PS: Pyriform sinus; PC: postcricoid; PW: posterior wall; TNM: tumor/node/metastasis.

**Table 4:** Swallowing function excluding radiotherapy in each group.

		Free Jejunal Flap Group		Cutaneous Free Tissue Flap Group		*p*-Value
		N = 154		N = 30		
		N	%	N	%	
Diet at discharge	FOIS 1-6	106	69	13	43	0.01
	FOIS 7	48	31	17	57	
Nasopharyngeal reflux	No	144	94	29	97	0.99
	Yes	10	6	1	3	
Anastomotic stricture	No	135	88	28	93	0.53
	Yes	19	12	2	7	

FOIS: functional oral intake scale.

**Table 5:** Odds ratios (ORs) and 95% confidence intervals (CIs) from logistic regressions of diet at discharge excluding radiotherapy.

	Diet at Discharge		
		Multivariable Analysis	
	N	OR (95% CI)	*p*-Value
Age			
<70	86	0.73 (0.38–1.40)	0.35
≧70	98		
Sex			
Male	165	0.53 (0.19–1.45)	0.22
Female	19		
Medical history			
Diabetes	32	1.04 (0.45–2.42)	0.93
No Diabetes	152		
Coronary artery disease	16	1.49 (0.46–4.82)	0.50
No coronary artery disease	168		
Cerebrovascular disease	13	1.35 (0.37–4.86)	0.65
No cerebrovascular disease	171		
T (TNM classification)			
½	32	1.06 (0.46–2.49)	0.89
¾	152		
Preoperative therapy			
Neo-adjuvant Chemotherapy	22	0.83 (0.32–2.19)	0.71
No neo-adjuvant Chemotherapy	162		
Reconstruction material			
Free jejunal flap	154	0.31 (0.14–0.72)	0.01
Cutaneous free tissue flap	30		

PS: Pyriform sinus; PC: postcricoid; PW: posterior wall; TNM: tumor/node/metastasis.

**Table 6:** Prevalence of pharyngocutaneous fistula in each group.

		Free Jejunal Flap Group		Cutaneous Free Tissue Flap Group		*p*-Value
		n = 188		N = 35		
		N	%	N	%	
Pharyngocutaneous fistula	No	170	90	32	91	0.99
	Yes	18	10	3	9	
Pharyngocutaneous fistula with operation		3	2	1	3	0.99

**Table 7:** Prevalence and type of post-operative complications in each group.

	Free Jejunal Flap Group		Cutaneous Free Tissue Flap Group		*p*-Value
	N = 188		N = 35		
	N	%	N	%	
Donor site complications					
Ileus	16	8	0		0.08
Infection	6	3	0		0.59
Hemorrhage	0	0	0		(-)
Neuroparalysis	0	0	0		(-)
Total	22	12	0		0.03
Systemic complications					
Respiratory disease	5	3	2	6	0.30
Cardiovascular disease	2	1	1	3	0.40
Others	11	6	2	6	0.99
Total	18	10	3	9	0.99
Cervical complications					
Fistula	24	13	6	17	0.43
Flap necrosis	9	5	1	3	0.99
Lymph fistula	26	14	3	9	0.59
Hemorrhage	5	3	0	0	0.99
Infection	26	14	3	9	0.59
Total	68	40	10	31	0.44

**Table 8:** Reported prevalence of postoperative pharyngocutaneous fistula.

Author	Year of Publication	Pharyngocutaneous Fistula			
		Free Jejunal Flap Group		Cutaneous Free Tissue Flap Group	
		N	%	N	%
Yu et al. [8]	2010			91	9
Lewin et al. [13]	2005	31	1	27	7
Huang et al. [14]	2015			45	11
Kurita et al. [15]	2018	243	3.7		

## Data Availability

The data that support the findings of this study are available from the corresponding author upon reasonable request.
